# Clinical Studies with Large Non-Emetic Doses of Ipecacuanha: With a Contribution to the Therapeusis of Cholera

**Published:** 1875-04

**Authors:** Alfred A. Woodhull

**Affiliations:** Assistant Surgeon U. S. Army


### CLINICAL STUDIES WITH LARGE NON-EMETIC DOSES OF IPECACUANHA: With a Contribution to the Therapeusis of Cholera.

(Continued from page 721 of March number.)

By ALFRED A. WOODHULL, M.D., Assistant Surgeon U. S. Army.

*A Special Report to the Surgeon General U. S. Army, read before the Atlanta Academy of Medicine.*

The preceding pages give in detail my own recent experience, with a *resume* of the modern literature of ipecacuanha in dysentery. There are many interesting points thereby suggested, which my field of personal observation has not been wide enough to embrace. But for a consideration of some of them, and for a pursuit of the subject in one direction, attention is invited to the remainder of this paper.

While engaged in these observations, and in the reflections to which they gave rise, and being led up, as it were, by the cases developing under my eyes, to employ ipecacuanha in large non-emetic doses in cholera-morbus, its possible usefulness in Asiatic cholera occurred to me. Aware that abstract reasoning not fortified by clinical experience at every step is seldom trustworthy in medicine, I have nevertheless felt it a duty, in view of no treatment having yet secured a fair degree of success, to expose the train of thought that leads to the supposition that this drug may prove useful in that disease. I do this with diffidence but not deprecating legitimate criticism, and with less reluctance because, at the worst, it will only add one innocent failure to the long list of unsuccessful trials, while it may develope the germ of cholera-control.

To explain my belief involves a recapitulation of the pathology of cholera, and something more; and I therefore embrace the opportunity to discuss, at the same time, the possible mode of operation of ipecacuanha in dysenteric and other affections. And although I can fairly claim the proposition as original, my subsequent studies have brought me upon indications, notably Dr. Waring's (*op. cit.*, p. 361,) which, although not identical, point in the same direction. I conceive that priority has little precedence over originality as a claim to credit, and that both are of small importance when compared with efficiency in the alleviation of disease. Those hints to the same end that I have found are faithfully recorded, and I only ask a careful reading of both the practical and theoretical parts of this paper.

It is not practicable even to enumerate here all the conceits that have been put forth to account for the phenomena of Asiatic cholera, but a brief notice may be taken of one advanced a few years since, before more seriously taking up the probable explanation of the disease. The distinguished names of Dr. Geo. Johnson and Sir Thomas Watson have given to the poison-to-be-eliminated hypothesis a certain foundation, and have drawn to it an attention that its abstract merits might not command. This hypothesis is, in brief, that the cholera phenomena result from the introduction of a specific poison into the blood, where it rapidly self-multiplies and spoils certain blood-constituents which are then ejected through the mucous membranes of the alimentary canal; that the poison circulating in the blood excites contraction of the muscular walls of the minute pulmonary arteries, arresting or diminishing the flow of blood in the lungs, which is the essential cause of the cholera collapse; that the copious discharges express nature's efforts to throw off a noxious material, and really form, therefore, a necessary part of the process of recovery; and that if the pouring forth of the vascular excretion be checked, (as it possibly may by opium,) the risk of fatal collapse is thereby increased. They therefore advocate "the evacuant or cleansing practice," and propose not to excite increased excretion, but to facilitate the discharge from the mucous canal of matters lodged there. Gentle elimination is the key-word. But this practice (except when abused by being transformed into active purging,) seems to be really little more than abstention from active interference, and a trust that nature will effect a cure, as we know that she often does. Its greatest merit is that it leaves no room for and reprobates the opium and alcohol treatment, and it is actively good by its suppression of those mischievous agents. It appears, also, that unfortunate practical experience has aided theoretical reasoning to destroy the fabric. (Mr. Sedgwick, *Lancet*, Oct. 7, Nov. 11, 1871; *Am. Jour. Med. Sci.*, Jan., 1872, p. 252.)

All the other conceptions of cholera-treatment have rested upon the supposition that the cholera-poison must be combated or its effects be nullified within the tissues. They are diverse in their nature, but upon those who hold to views materially differing from what the profession at large has settled upon, rests the burden of proof. The current belief as to the nature and cause of cholera cannot be expressed better than in the summary of Professor Alonzo Clark, (*Med. Record*,* i., Nos. 2, 3, quoted by Burrall, *Asiatic Cholera*, p. 136,) viz: "It may be stated that there is a poison the exact nature of which is not perfectly understood; that this poison introduced into the system causes disturbance of innervation or a sort of paralysis of the ganglionic nervous system; that this leads to extensive hyperæmia of the alimentary canal, resulting in the symptoms described, and to the reflex phenomena aluded to, [referring to the lecture from which this is taken,] and, as the disease progresses, obtaining more or less an inflammatory character." Later researches all tend to confirm this view.

It is generally admitted that the first, or, the condition of painless diarrhœa, is the only stage of this disease in which therapeutics have heretofore been of avail; and there is fair reason to regard sulphuric acid, properly administered, as at least one remedy that is reliable in the control and perhaps in the prophylaxis of that condition.† Favorable experience with it is accumulating.‡

This watery-diarrhœaic condition, when not controlled, carries the patient into the second or cold and dangerous stage of cholera. Our knowledge of pathology is, unfortunately, too limited to explain exactly why the disease may sometimes be strangled while at others it developes into a fiercer type. But there is no reason to suppose that the condition which culminates in collapse is more than the legitimate development of the unchecked morbid agent. To employ a rude material figure, it may be likened to the leak in a levee made by an insignificant shell-fish; but when the water pours through in a flood, the crevasse itself is at once cause and effect, and must be cured by means so removed from the first simple but efficient remedy as to be virtually distinct in character. Neglecting, as probably visionary, the search for a cure that shall be an antidote as one chemical trans-

†"Its action in cholera is explained thus: The contents of healthy bowels are naturally acid; but in true choleraic diarrhœa the alkaline serum of the blood is poured out so copiously into the intestines as to render their contents no longer acid. Acids not only restore the natural acidity of the bowels, but cause the endosmotic current, which is always towards the alkaline side, to return to its proper course and thus re-establish the function of absorption." PETERS, *Notes on Asiatic Cholera*, 2d ed., 1867, p. 187.

‡STILLE, *Therapeutics*, 3d ed., i., p. 282; WARING, *Therapeutics*, 2d Am. ed., p. 566; CURTIN, *Phil. Med. Times*, iii., p. 649; H. C. WOOD, *Therapeutics*, p. 83.

forms another, we are required to find a treatment that will neutralize the cholera-poison in the sense, for instance, that opium and belladonna are antagonistic, or as alcohol counteracts snake-bite. Some one drug may possess this antagonism, or perhaps combinations will be necessary.

Preliminary to the therapeutic proposition, a *resume* of the pathology of the second stage is presented.

Serous discharges, usually profuse, accompanied by painful cramps and culminating in collapse, is a comprehensive symptomatic description of it. Not attempting to describe all the pathological appearances, we find in the abdomen, which by common consent bears the brunt of the disease, that: the small intestine has a peculiar rosy appearance, its mucous membrane is finely injected and often shows extensive ecchymoses, and the contents of the bowel appear reddened with blood; the mucous membrane and whole intestinal wall are swollen and relaxed from œdematous infiltration; the glands are generally swollen and distended; the villi lose their epithelium; and, in brief, in the 'height of the disease the characteristic changes consist chiefly in extensive catarrh of the intestines accompanied by detachment of the epithelium and copious transudation, in decided thickening of the blood, and excessive hyperæmia of the kidney.' (*Niemeyer;* Am. fr. 8th Germ. ed., 1869, ii., pp. 648–9.) We cannot look upon one particular lesion as embracing the whole pathology of the disease, for the interdependence of the physical functions upon each other and upon various anatomical elements is well recognized as complete and complicated. But it is fairly understood that preponderating though not exclusive influences are exerted over different organs by individual and varying agents. One of the most clearly defined of these, anatomically and pathologically, is the distribution to and control over the intestines of the sympathetic nerve. And it is generally conceded that the essentials of the pathology of cholera are found in its profound disturbance, although all writers are not agreed as to the mode.† An artificial derangement of the sympathetic is not

---

† See A. CLARK (*op. cit. sup.*); GREENHOW, cited in "BURRALL on *Asiatic Cholera*," 1866, p. 86); MAREY (*Gaz. Heb.*, Nov. 24, Dec. 1, 1865 quoted by BURRALL, p. 137); SEDGWICK (*Med. Chi. Trans.*, li., 1868, pp. 1-42 ; JEAFFRESON (*Edin. Med. Jour.*, Dec., 1866, p. 520); MEIGS and PEPPER (*Diseases of Children*, 4th ed., p. 386); PEPPER (*Phila. Med. Times*, i., p. 172); SEDGWICK (*Lancet*, Dec., 1871, p. 644).

cholera; but the most of the choleraic symptoms may be induced by its perturbation.

To recapitulate briefly the leading points connected with the sympathetic: We find; intense hyperæmia of the mucous membrane of the stomach and intestines follows extirpation of the cœliac plexus (Flint, *Physiology*, iv., p. 483, citing Samuel, *Jour. de la Phys.*, Paris, 1860, iii., p. 580); Moreau showed by a series of experiments that, in isolating three loops of intestine in a fasting animal and dividing all the nerves passing to the middle loop, it was found next day filled with clear alkaline liquid, colorless or slightly opaline, while those with the nerves intact were empty and the mucous membrane was dry (Flint, iv., p. 434, citing Moreau, *Bul. l'acad. Med.*, Paris, 1860 [?], xxxv., p. 388); these "observations on the influence of the sympathetic nerves upon the secretion of liquid by the intestinal canal, are peculiarly interesting in their bearing upon the sudden occurrence of watery diarrhœa" [and of cholera] (Flint, iv., p. 434); experiments of Payrani "show that the sympathetic has a remarkable influence over the secretion of urine. * * When the sympathetic is divided the quantity of urine and urea sinks to the minimum" (Flint, iv., p. 434, fr. *Comptes rendus*, Paris, 1870, lxx., p. 1300); "the rice-water flux * * may occur also in other [than cholera] cases in which, as in cholera, there is a neuro-paralytic condition of the digestive canal" (Sedgwick, *Lancet*, Dec., 1871, p. 644). Besides the foregoing established facts, there is a generally acknowledged, but not yet clearly explained, connection between the function of perspiration, physiological and pathological, and the influence of the sympathetic. (See, particularly, a valuable paper by W. Pepper, *Phila. Med. Times*, i., p. 173; also Flint, iii., p. 138, *Rendu Arch. Gen. Med.*,* Sept., 1869, p. 286; Bartholow, *Am. Jour. Psych. Med.*,* Jan., 1869, p. 134; Ogle, *Med. Chir. Trans.*,* lii., 1869, p. 151). Condie remarks (Peters, *op. cit.*, p. 78,) that in cholera "this copious perspiration is generally not dwelt upon with sufficient emphasis, for it is a source of great exhaustion; it sets in early in the attack and becomes excessive towards the close of fatal cases."

It is, however, probable that the sympathetic ganglia are not independent nerve centres, and therefore the spinal nerves are likely to be implicated. It is also probable that the pneumogastric, especially, is involved in the general nervous disturbance of this disease, for its branches to the small intestine are very numerous, (Kollmann; Flint, iv., p. 211;) and it could hardly escape being involved either directly or reflexly; but exactly what part it plays has not been clearly made out.

To the foregoing I add other explanatory quotations, for I have nothing new to present on the pathology of the disease. And these, with what has already been noted, complete the pathological description of the second stage. Jeaffreson, writing "On the Pathology of Cholera Collapse," (*Edin. Med. Jour.*, 1866, xii., p. 1,) asks, partly in criticism of Dr. Johnson's hypothesis and partly in support of the idea that the intestinal irritation is the cause of the collapse, "If this state [i.e., the intense intestinal congestion of collapse] were produced by a special poison acting upon the nerve centres, even, which regulate the supply of blood in the arteries generally, how does it happen that the nerves and arteries of the intestines are either exempted from the peculiar influence or are acted upon in just the reverse manner; the small intestines (and in a less degree the large intestines) being the only portion of the body to which an active determination of blood has occurred?" (p. 530.) This question finds a positive answer in Flint's words, where he discusses the experiments of Cyon on the depressor nerve: "We are sufficiently familiar with reflex paralysing action upon the blood-vessels through the sympathetic system; and when we call to mind the immense extent of the abdominal vascular system we can readily understand how, if the resistance to the flow of blood be diminished by paralysis of the muscular coats of the small arteries, the pressure in the large arteries would be reduced" (iv., p. 232).

Dr. Jeaffreson, in the same article, describes the whole abnormal condition thus: "The chain of causation appears to be the following: A poison in the alimentary canal acts there as a direct irritant, causing more or less rapidly-developed congestion and inflammation of the whole small intestine, to which much blood is determined. The intestine, meaning by the term the tissue of the various coats, becomes full and turgid, and acutely œdematous, whereby a strong rapidly-developed impression, resulting in shock, is made upon the innumerable and widely-spread branches of the sympathetic from the solar plexus by which the duodenum, jejunum and ileum are supplied. [I do not understand that the shock is necessarily or probably due, as Dr. Jeaffreson expresses it, to afflux of blood, but that, rather, the afflux follows the paralysis of the abdominal sympathetic from some toxic principle directly affecting it.—A. A. W.] The well-known intimate connection of the solar plexus with the splanchnic and pneumogastric nerves, and also with the posterior roots of the corresponding spinal nerves, insures the diffuse spread of this impression, amounting to a shock, from which results a general slow contraction of the organic muscular fibres of the whole arterial system, affecting not only the pulmonary artery but the systemic arteries, including those of the kidneys and spleen, which are found anæmic after death, and also including, in all probability, the hepatic artery, though, from the peculiar nature of the circulation in the liver, the effects there are less manifest" (p. 531).

Mr. Sedgwick, in a very valuable article 'On Some Analogies of Cholera in which Suppression of Urine is not accompanied by symptoms of Uræmic Poisoning,' (*Med. Chir. Trans.*, li., 1868, p. 1,) very clearly shows that the ingestion of certain substances which might be presumed to cause blood-poisoning, such as decaying animal and vegetable matters, milk that has undergone some morbid change, various fungi, but also violent irritants,† as excessive doses of arsenic, nitric acid and other corrosive poisons, and further, many severe accidents and incidents attending on disease, as wounds of the abdomen, and lacerations and perforations of the stomach and upper intestines, induce a collapse that in no essential respect can be distinguished from that of cholera. He says: "There will not, however, be much difficulty in recognizing, on further inquiry, that, whether the mischief be the result of perforation or of obstruction of the small intestine, the suppression of urine which results from it must be regarded chiefly as an indication of the intensity of the collapse, consequent on the comparative suddenness of the mischief, and its nearness to the abdominal centre of the sympathetic nervous system; in the same way that a corresponding suppression occurs in severe cases of cholera, whilst in mild cases of this disease which it has been customary to refer to as choleraic diarrhœa, there may be, and usually is, the characteristic flux without any suppression of urine" (p. 27). Among other contingencies in which the collapsed condition with urinary

† To speak of '"irritants," "irritating substances," etc., in the bowels,' often misleads. Usually irritation is looked upon as akin to stimulation; but as used in the text the facts will be quite as well understood by describing them as 'paralysing,' which is the state of the case as far as the nervous system is concerned.—A. A. W.

suppression occurs is intestinal obstruction, and it has generally been accounted for by the degree of peritoneal inflammation or the amount of vomiting: but Mr. Sedgwick points out "that any correlation which may exist between vomiting and suppression of urine in these cases appears to be simply the result of both conditions being primarily dependent on the affection of the abdominal nervous system" (p. 34). Attention is particularly invited to this opinion, as consonant with the original views to be expressed later in this paper. And in reply to those authors who suppose "that the suppression of urine, if not the collapse itself, is essentially dependent in cases of cholera on the flux from the stomach and bowels," he asserts that "not only is there ample evidence derived from direct observation to refute it, in addition to the evidence which has been adduced from analogy, but that there is, moreover, a self-destructive fallacy in thus supposing that the digestive canal could serve for the complete escape of excrementitial matter usually discharged by another outlet, whilst the elimination of excrementitial matter proper to the canal itself is completely checked. For just in the same way that jaundice results from the increasing accumulation of the essential elements of bile in the blood, and uræmic poisoning from the increasing accumulation of urea, so the absence of these two morbid conditions shows that during collapse there is no excess of excrementitious matter for either the kidneys or the liver to eliminate, and therefore no urine is conveyed to the bladder and no bile to the alimentary canal" (p. 41).

We may well agree with Mr. Sedgwick that "although analogy can only be referred to for the purpose of supplying indirect evidence, yet, on the present occasion, this is so strongly in favor of cholera being primarily due to an affection of the sympathetic nervous system developed through the medium of the digestive canal, as scarcely to need any further evidence to support it" (p. 42). And we may credit the statement of Dr. Davey, whom he quotes, that "the fatal depression in cholera, consisting in the complete annihilation of the action of all the vital organs, may be at any time simulated by pressing the solar ganglion on the fore part of the bodies of the vertebræ over which it lies" (p. 43). And, as Mr. Sedgwick remarks in a postscript, important evidence in support of that argument has been supplied by M. Moreau's experiments (already cited) on section of the abdominal nerves in relation to intestinal flux.

When death does not occur in the condition of collapse, the stage of reaction sets in. This is generally admitted to be a febrile state that is to be treated on general principles, bearing in mind both the nervous prostration lately undergone and the possibility of reinducing it by want of care.

This recapitulation, containing nothing that is original or has not been published before, may seem superfluous; but it is made a part of this paper for convenience of reference.

We have now reviewed the experimental use of ipecacuanha in bowel affections, and have presented an outline of the pathology of Asiatic cholera as at present held. It remains to bring the two into efficient accord.

The present state of medical science does not permit us to generalize nor even to frame a plausible hypothesis upon the correlation of diseases, except in a very limited and doubtful degree. But neither does it allow us to consider them as isolated foreign entities, that may be implanted or plucked out of the human system as integers. There is often an undeniable blending, and the distinction between members of many well-marked groups is frequently one that implies affinity as well as diversity. Thus, erysipelas and puerperal fever, diphtheria and scarlet fever, are examples of such rudimentary consanguineous grouping. These collections of symptoms, fusing with more or less completeness, connect pathological classes that in appearance are quite distinct. And the real object of this portion of this paper is to invite attention to the presumed benefits that will accrue from the employment in epidemic cholera of ipecacuanha, which is known to be strikingly useful in other diseases that, in my opinion, may claim kinship with that pestilence. This will be attempted by the citation of authentic cases and by quoting statements and opinions of reliable observers in illustration of the different steps of the problem.

The first, reported by Mr. John Higginbottom (*Lancet*, 1845, i., p. 732,) under the title, "Ipecacuanha, in Emetic Doses—as a Powerful Restorative in some cases of Exhaustion and Sinking," is reproduced in detail on account of its intrinsic interest, and from the comparative inaccessibility of the original. He says: "In the year 1814, I was first led to see the extraordinary beneficial effects of ipecacuanha as an emetic, in a female forty years of age, who was in a sinking state in the last stage of cholera

[morbus]; her countenance was shrunk, extremities cold, cramp in the legs, and other symptoms of approaching dissolution. I had previously attended two similar cases, where I had given opium, brandy and medicinal cordials, and both patients died. I was induced, in this instance, to give a scruple of ipecacuanha, from having frequently seen the good effects of it in the early stage of the disease. After the lapse of two or three hours, I again visited my patient, fearing I should find her dead, but, to my great pleasure and surprise, so great a change for the better had taken place as to appear almost incredible; the whole of her body was of a natural warmth, the dangerous symptoms had disappeared, and she made no complaint, except that she was very weak. She had no further unfavorable symptom of the disease, and was soon convalescent. [Compare with cases VI. and VIII. *ante.*] My confidence in the ipecacuanha, as a remedy in such cases, has now [1845] been confirmed during the practice of thirty years; the purging, vomiting and cramp often entirely cease after the emetic operation of the ipecacuanha, but I have thought it proper to give, in about two or three hours after the emetic, a pill, with a grain of opium and five grains of blue pill, to allay any remaining irritation of the stomach and intestines, and an aperient with one scruple cf rhubarb and two of the sulphate of potash, to assist the natural action of the bowels, and a simple saline effervescent draught every two or three hours afterwards; weak tea, well-boiled gruel, milk, with sago or arrow-root as nutriment, and diluents." This case appears to be typical, and was reported not as being unique but as an example.

In the same paper Mr. Higginbottom cites the case of a lady who was sinking rapidly from post partum hæmorrhage in 1827, whom he had previously attended in a similar condition in 1821 and 1823, using then various remedies; and having observed that in the former instances "there was no amendment until she had ejected the contents of the stomach," gave her half a drachm of ipecacuanha as an emetic. A full vomiting followed and "the hæmorrhage ceased directly, and did not return."

He also gives the case of a very delicate lady, aged 23, who, undergoing a severe labor with puerperal convulsions (for which twenty ounces of blood was taken), and embryotomy also having been performed, "was exceedingly low afterwards." His record reads: "About the ninth day she complained of severe pains in the course of the colon, particularly at the caput coli and the sigmoid flexure." "Mustard plasters were applied and active purgatives, with benefit, but a continued vomiting came on attended with considerable lowness. Dr. Hutchinson was called in to visit her with me. Injections of half-a-pint of beef-broth with half an ounce of turpentine were administered every four hours; a common blister of cantharides was applied to the scrobiculus cordis; plain gruel and other light nutriment was given, as most likely to remain on the stomach. The vomiting still continued; the turpentine injections occasioned much pain after they were administered, and there was an alarming increase of exhaustion and sinking. In this case it occurred to me that an emetic dose of ipecacuanha was the most probable remedy to rally the sinking powers and, with the concurrence of Dr. Hutchinson, I gave half a drachm and remained with her during its operation. A fuller vomiting occurred than I could have expected although it was small in quantity, yet it occurred to me that the natural effort had long been exerted in vain to accomplish what the ipecacuanha directly effected—that of completely emptying the stomach. I remained with my patient an hour, and left her somewhat better. After I had gone she turned herself on the left side and remained so still for several hours as to alarm her husband, who sent for me directly, fearing she was dying. I found her pulse much improved; she was still lying on her left side; the sickness had abated." She rapidly and fully recovered.†

It is ungracious to criticise a record so valuable, but it appears to me that the action of the drug and, in a degree, the condition of the patients have been misinterpreted by the reporter. In the first, or cholera morbus case, which is set up as a precedent "confirmed during the practice of thirty years," it is probable that the use of the drug "as an emetic" was an unnecessary exhibition of its powers. If the same quantity had been given with the precautions that now are taken, it is altogether likely that the same effect, without the vomiting, would have been attained. It seems to have been given in that case as a last resort, because emesis often relieves the disease in its earlier stages by

† Duckworth states (*St. Barth. Hosp. Rep.* vii., p. 121,) that Mr. Higginbottom (*Brit. Med. Jour.*, Feb. 1869,*) reports cases of post partum flooding "where it checked the bleeding after ergot of rye had failed." He adds, however, (p. 122,) that Mr. Higginbottom also reports a case of flooding "at once checked by the vomiting induced from the irritation of the fauces by a feather."

removing the irritating ingesta which may then be the exciting cause. But here the conditions were wholly different, for she "was in a sinking state in the last stage of cholera." Nor is there any record of the ejection of such offending matter. How far the succeeding medication of opium, blue pill, aperients, saline effervescents, etc., may be necessary must depend upon the individual cases. But even in Mr. Higginbottom's opinion they are but secondary and incidental.

In the second, or the post partum hæmorrhagic case, it is not at all clear that the vomiting *qua* vomiting was the cause of the improvement. It seems to me that here the *propter hoc* and the *post hoc* have again been confused. A reason why improvement and recovery followed, and how the ipecacuanha and not the vomiting may have been the efficient cause, will be presented later.

In the third case it really appears that the emesis had nothing to do with the cure, for that function had repeatedly been exercised without benefit. As the case progressed, it is recorded, "a continued vomiting came on, attended with considerable lowness." This was clearly a pathological condition and it was chiefly to relieve it, the sign of marked vital depression, that the later efforts of her physicians were directed. There is no history of irritating ingesta here, and to suppose that the pathological vomiting was cured by the artificial vomiting would be the purest homœopathy. Moreover, it is expressly noted that, although it was fuller than could be expected, "it was small in quantity." I must, in this case also, regard the vomiting as incidental. It, further, seems to me that the puerperal condition† of this patient is chiefly significant in that it damaged her general health and depressed the sympathetic, and that the suffering in the colon, which has made no special impression on the reporter, and the intestinal disturbance generally, bring the case into direct relation with some of those that make up the original reports of this paper.

More than twenty years later (in 1868) Mr. Higginbottom read before the British Medical Association a paper upon "Ipecacuanha in Emetic doses as a Stimulant, Restorative, Eliminative, and Adjuvant, in various cases of Disorder and Disease." I have been unable to consult the original memoir, but it is thus somewhat vaguely epitomized; (*Brit. Med. Jour.*, 22d Aug., 1868;

†See Trousseau's views, to be noted presently.

*Rankings' Abst.*, xlviii., p. 92.) "The author inferred that the interests and advancement of the profession could not fail to be greatly promoted by a long, careful, and practical investigation of a single therapeutic agent. He was of opinion that emetics were much less used than formerly. Ipecacuanha, besides its specific properties as an emetic, expectorant and diaphoretic, had other valuable properties which he believed had not been particularly noticed by the profession. He had constantly, for more than half a century, administered ipecacaunha in English cholera, fevers, erysipelas, neuralgia, periodical drunkenness, uterine hæmorrhage, complaints in old age, syncope, senilis, etc. The main efficacy of ipecacuanha is in stimulating and restoring the normal action of the capillary system."

Although the notion of necessary emesis is very manifest in this abstract, the last sentence contains the kernel of his fifty years' experience—its main efficacy is in stimulating and restoring the normal action of the capillary system.

Further teaching as to the power of this drug may be found in a Clinical Lecture on Parturition, by Tyler Smith (*Lancet*, 1848, ii., p. 658). "2. Ipecacuanha is another medicine which is sometimes given in uterine hæmorrhage. This medicine, by its emetic action excites contraction of the abdominal muscles and compression of the uterus, which in turn may re-excite some amount of uterine reflex action, but over and beyond this it appears to have a special action upon the uterus, increasing its contractile power beyond what we could imagine to occur from the merely secondary effects of vomiting. Ipecacuanha, then, appears to influence both the medulla oblongata and the lower medulla spinalis. This double action of ipecacuanha upon the extremities of the spinal centre is very extraordinary." This is a direct recognition of its possession of virtues beyond, if not independent of, its emetic quality.†

In internal hæmorrhages generally, Mr. Trenor (Waring, *op. cit.*, p. 360; fr. *Dublin Jour.*,* xviii., p. 481,) "gives it in such

† Doulcet and Desormeaux used it successfully in epidemics of puerperal fever in 1782. Trousseau and Recamier employed it in all the conditions of puerperal origin, regardless of their cause or nature, and always with benefit. Polichronie, citing these facts, (*op. cit.*, pp. 20-21,) attributes the good results to the relief of the gastric embarrassment [by emesis]. Trousseau asserts that 'nearly all the complications [*accidents*] that accompany the puerperal state are charmed away by ipecac.' (*Considerations sur l'Ipecacuanha en Medicine.* These par V. Decugis. Montpellier, 1866, p. 45.)

doses as to produce nausea, without actual vomiting; and this procedure was attended with marked benefit, arresting the hæmorrhage, and restoring heat and life to patients who were in a state of collapse from excessive loss of blood."

Dr. Osborne gives in uterine hæmorrhage and menorrhagia twenty grains of the powder "in the evening, followed by an acidulated draught in the morning. The discharge usually ceased in twenty-four hours; and if a relapse occurred, a repetition of this emetic never failed to render the cure permanent." (Waring, *op. cit.*, p. 360; fr. *Trans. Irish Col. Phys.*,* v., p. 18.)

If it is a mere evacuant, it is contradictory to attach to it such attributes.

Very weighty testimony to the hæmostatic property of the drug is given by Trousseau (*Clinical Medicine*, Eng., fr. 3d Fr. ed., i., p. 540,) where, treating of hæmoptysis, he says: "When the parenchymatous hæmorrhage is obstinately recurrent ipecacuanha [in small doses] is a remedy which seldom fails. I am not at present referring to ipecacuanha administered as an emetic, which is more to be relied on in what is called bronchial hæmorrhage." He then cites a case of the latter sort where a patient "twice within the space of six months had frightful hæmoptysis: twice it was immediately arrested by four grammes (rather more than a drachm) of powder of ipecacuanha, administered within the space of half an hour in such a way as to cause violent vomiting." He also details several other cases with the same result and proceeds: "Should, however, there be a relapse of the hæmoptysis, the use of the ipecacuan must be resumed. I never hesitate in such circumstances to return to it two or three times, if necessary, and I have never seen the least inconvenience result from the proceeding. Gentlemen, this is not a new method of treatment. For the last two centuries, physicians have lauded the Brazilian root as a remedy in all forms of hæmorrhage; and Baglivi [1696] says: '*Radix ipecacuanhæ est specificum et quasi infallibile remedium in fluxibus dysentericis, aliisque hæmorrhagiis.*' Nevertheless, gentlemen, the hand trembles when it administers this remedy for the first time in the hæmoptyses. We are accustomed to prescribe the greatest quietude to our hæmoptoic patients: we counsel them to keep absolute silence: we tell them to restrain the slightest effort to cough: the very most we allow them to do is to breathe, and so frightened are we for congestion, even passive congestion of the lung, that we act as if we placed them in peril by permitting them to make the slightest effort. Yet here we are giving a medicine which produces vomiting, during which the face swells, the blood stagnates in the veins by which it is being conveyed to the auricles: and consequently, the pulmonary veins become distended. One might expect that such treatment would cause the hæmoptysis to return in a much more profuse degree; but in place of this, it is stopped in nearly every case. Here is one proof more of the small reliance to be placed on theoretical explanations, and of the value of empirical facts, without which, indeed, therapeutics would be a nullity."† It may not, perhaps, be presumptuous to suggest that the time is approaching when theory, as well as empiricism, will support the practice.

The latest clinical record as to this feature of that medicine that has fallen under my notice is by Dr. William Martin (*New York Medical Journal*, xiv., 1871, p. 177,) who reports a case of wound of the right tonsil, to the apparent depth of two-thirds of an inch, by a bamboo pipe-stem. No serious immediate hæmorrhage occurred. Twenty-one hours afterward there was "positive jutting arterial hæmorrhage from back and upper surface of the wound which altogether presented a most discouraging and alarming appearance." There was general tumefaction, headache in the region of the lateral meningeal arteries, and the patient was "in a state of prostration and fear." There were prescribed twenty grains of chloral, and a gargle containing one part in four of muriated tincture of iron. Nine hours afterward there was no change except suffocation was complained of, and Dr. Martin writes "(and being *quite certain* that hæmorrhage was not from the carotid) I gave two-grain doses of ipecac. every hour, till third dose caused *gentle vomiting*, and with it immediate contraction of the tonsil (thus lessening wound) and causing hæmorrhage at once to stop." The bleeding did not recur and no other hæmostatic was used. Dr. Martin adds these remarks: "* * I am quite convinced, from the experience of nineteen years, that the theory and practice of my lamented teacher, Sir William Lawrence, Bart., that in such cases where capillary hæmorrhage occurs and the position of the main artery dangerous, that after the administration of *small doses* of ipecac., until

†Duckworth (*St. Barth. Hosp. Rep.*, vii., 1871, pp. 117-121,) cites an interesting collection of authorities on this point, and reports three successful cases of his own where small doses were used.

a *gentle* vomit is caused, natural plugging follows, contraction of tissues and safety is insured, for in such cases and under such treatment muscular contraction *never* fails. I have tried it many times (even so late as last week) in obstinate contraction [?] of uterus and never experienced ill effects."

In his lately published papers, (*Prog. Med.*, Nos. 12, 16, 25, 28, 29, 30, 1874,) and this is a very interesting confirmation of the views herein expressed, M. Chouppe reports the successful use of ipecacuanha in ten out of twelve cases of excessive perspiration in phthisis. See also Polichronie (*op. cit.*, pp. 46–50.)

We have now collected sufficient testimony to illustrate the possession by ipecacuanha of peculiar powers, and powers that are not generally appreciated. It is evident that the incredulity with which the accounts of its extra-emetic properties are generally received, is largely due to the want of a plausible theory broad enough to cover the apparently antagonistic conditions. It remains, then, to offer a reasonable explanation for the reconciliation of some seeming discrepancies, and to present an hypothesis that may assist in the treatment of the cholera. And in this matter the latest systematic writer (H. C. Wood, *op. cit.*, pp. 364–5,) gives us license for speculation when he announces *ex cathedra* that "its physiological action is not, as yet, well made out" and "it is evident that until further studies are made it is impossible to frame any accurate theory as to the action of ipecacuanha."†

†The following are the views of some of the later students as to the action of this drug:

Trousseau and Pidoux regard it as a styptic when used in internal hæmorrhages (Duckworth, *St. Bart. Hosp. Rep.*, v., 1869, p. 220).

Pecholier (*Recherches experimentales sur l'action physiologique de l'ipecacuanha*, par G. Pecholier, Professeur, etc., 1862,) explains (p. 7) its reputed benefit in many diseases by its emetic and evacuant effects in those which are complicated with gastric disorder. He finds it very depressing (*hyposthenisante tres-prononcee*) upon rabbits and frogs, and considers its legitimate action to be temporarily depressant upon the nervous system, the ephemeral duration depending on its elimination (pp. 47–9). In some cases there is a temporary reaction after the severe depression (p. 51). Its good effect in pneumonia is due to its revulsion upon the intestine (p. 53). He believes that its irritant effect is so great as to render it hurtful in true gastritis and enteritis, but that its usefulness in dysentery arises by the revulsion it induces from the large to the small intestine (p. 39). He claims (p. 50,) Giacomini and the whole Italian school as agreeing with him in classing it among the counter-stimulants.

The common understanding of the action of this drug, in other than very small doses, is that it vomits. Ipecacuanha and emesis are as synonymous, popularly, as opiate and anodyne, but that it is not inevitably an emetic, there are fifty proofs in this single record. It is notorious that the nausea of ipecacuanha, when vomiting does occur, is of short duration and is not intense. To diminish the force of the circulation, to deplete, to locally irritate the stomach, which are the modes of action attributed to it by those who hold that its curative action in dysentery depends on emesis, all would be much better accomplished by other agencies than this; while the *coup de grace* to the emetic idea is given by the fact that the less the emesis the more effectual is the treatment. Any one who has seen all the characteristic and painful symptoms of dysentery subside in a few hours, and commonly without vomiting, under a full dose of this drug, would neither anticipate nor obtain similar results from any emetic as such.‡ As we have already seen, Dr. Tyler Smith, twenty-five years ago, ascribed to ipecacuanha a peculiar power over "the lower medulla spinalis" entirely independent of its emetic properties, which he attributes

---

Duckworth (*op. cit.*, v., p. 222,) supposes that emetia excites the vaso-inhibitory filaments of the vagus, resulting in inaction of the motor branches and a condition of paralysis or passive dilatation of the blood-vessels presided over by this nerve. But the same writer also says of it (vii., p. 100,) "an irritant action may directly excite the vaso-motor centre [medulla oblongata?] and so cause increased contraction of the smaller arteries and possibly of the capillaries" either directly or by reflex action through the vagus. These suppositions appear to attribute directly opposite properties to the drug.

Chouppe (*Progres Medical*, No. 29, 18th July, 1874,) says: Ipecac. absorbed by any channel seems at the very moment of its absorption to produce an anæmia with dryness of the intestinal mucous membrane; perhaps if the action of the medicine is prolonged, it is eliminated more abundantly by the gastro-intestinal mucous membrane (which is not yet irrefutably proved) than by the other emunctories, and it may produce inflammation and hæmorrhages. He acknowledges (No. 30,) the great difficulty of determining how it acts in the profuse sweating of consumption, but suggests that it may be through the vaso-motor system.

Polichronie (*op. cit.*, p. 97,) concludes that no vaso-constrictor action is exerted by it, but that it diminishes arterial tension, and it probably induces a revulsive action which results in inflammation of the mucous membrane of the intestine; and that it acts in the diarrhœas by substituting for the pathological inflammations an open (*franche*) inflammation which spontaneously tends to recovery. He thinks it checks profuse sweating either by being eliminated through the sudoriporous glands, thus drying up (*tarir*) their secretion, or by its revulsive intestinal action.

‡ Nor from any substitutive intestinal inflammation, however set up.

to its action upon the medulla oblongata. It seems to me, speaking modestly, that emesis is one of its accidental and non-essential qualities.

Now while the dynamics of vomiting, the mechanical operation of the function, is fairly understood, its essential cause or causes are yet obscure. Flint observes (*Phys.*, iv., p. 306,) "It is undoubtedly induced by causes which operate through the nervous system * *. Irritation of the sympathetic nerves, particularly of the abdominal ganglia, will produce vomiting. * * there are many avenues for the passage of these impressions to the nervous centres. * *. The action of emetics which operate through the blood * * is probably induced by the direct impression made by these substances on the nervous centres." Professor Carson, discussing the action of emetics, says (*Phila. Med. Times*, ii., p. 344): "In many cases a state of exhaustion or loss of nerve-generating force is at the foundation of this excessive susceptibility to impressions that occasion vomiting." "This is seen in the sickness of stomach attendant on the loss of blood." "Disagreeable sights, odors or tastes, or even recollections of them may affect the brain sensationally and operate in the same way." This, we are to presume, occurs by reflex action on the sympathetic, as in like manner fear sometimes causes intestinal or cutaneous relaxation. Now, although the incidents that are the precursors of ordinary vomiting are well enough known, I, for one, have in my own mind no perfectly clear conception of the exact method in which ipecacuanha exerts its various powers, powers that are remarkable if not unique. But we may imagine, for illustration's sake, the nervous influence in health to be represented as in a state of equipoise, and that as it is deranged in one direction or the other emesis will occur. In this view ipecacuanha instead of being a sedative, as it is so often styled, is a sympathetic (ganglionic) stimulant that exalts one side of the beam and with the disturbed balance tonic vomiting, so to speak, occurs. The vomiting of irritability or exhaustion, on the other hand, results from the depression of the beam, and its equilibrium is restored by the positive influence of the drug. A more comprehensible comparison may, perhaps, be found, in what we know relative to the human temperature. When that is below a certain point we are ill; the addition of heat carries it up to the norm and we are well; but the same degree of heat added in health creates illness. Figures at best are imperfect guides and these are very rude, but it is only by some such comparison that I can bring myself to understand how our ordinary experience with the drug can be reconciled with its well-established control over the morning-sickness (irritability) of pregnancy, with its influence in certain forms of atonic dyspepsia, and with (as I believe) its efficacy in the vomiting of cholera morbus and allied diseases. It seems to me that we must either fall back upon some such notion, or must suppose that there is another principle besides and antagonistic to the well-known emetia, the reputed and generally-recogned essence of the drug.

*(Concluded in next number of Journal.)*

